# Variation and oscillation for the multilinear singular integrals satisfying Hörmander type conditions

**DOI:** 10.1186/s13660-018-1689-8

**Published:** 2018-04-20

**Authors:** Yinhong Xia

**Affiliations:** School of Mathematics and Statistics, Huanghuai University, Zhumadian, P.R. China

**Keywords:** 42B25, 42B20, 47G10, Variation operator, Oscillation operator, Multilinear operator, Hörmander condition, Singular integral, BMO

## Abstract

Suppose that the kernel *K* satisfies a certain Hörmander type condition. Let *b* be a function satisfying $D^{\alpha}b\in BMO(\mathbb{R}^{n})$ for $\vert \alpha \vert =m$, and let $T^{b}=\{T^{b}_{\epsilon}\}_{\epsilon>0}$ be a family of multilinear singular integral operators, i.e.,

$$\begin{aligned} T^{b}_{\epsilon}f(x)= \int_{ \vert x-y \vert >\epsilon}\frac{ R_{m+1}(b;x,y)}{ \vert x-y \vert ^{m}}K(x,y)f(y)\,dy. \end{aligned}$$

The main purpose of this paper is to establish the weighted $L^{p}$-boundedness of the variation operator and the oscillation operator for $T^{b}$.

## Introduction and results

Let *K* be a singular kernel in $\mathbb{R}^{n}$ and satisfy

1 $$\begin{aligned} \bigl\vert K(x) \bigr\vert \leq\frac{C}{ \vert x \vert ^{n}} \quad\text{for }\vert x \vert >0, \end{aligned}$$

where *C* is a fixed constant. Consider the family of operators $T=\{T_{\epsilon}\}_{\epsilon>0}$, where

2 $$\begin{aligned} T_{\epsilon}f(x)= \int_{ \vert x-y \vert >\epsilon}K(x-y)f(y)\,dy. \end{aligned}$$

The *ρ*-variation operator is defined by

$$\begin{aligned} \mathcal{V}_{\rho}(T) (f) (x)=\sup_{\epsilon_{i}\searrow0 }\Biggl(\sum _{i=1}^{\infty}\bigl\vert T_{\epsilon_{i+1}}f(x)-T_{\epsilon_{i}}f(x) \bigr\vert ^{\rho}\Biggr)^{1/\rho}, \end{aligned}$$

where the supremum is taken over all sequences of real numbers $\{ \epsilon_{i}\}$ decreasing to zero. The oscillation operator is defined by

$$\begin{aligned} \mathcal{O}(T) (f) (x)=\Biggl(\sum_{i=1}^{\infty}\sup_{t_{i+1}\leq\epsilon _{i+1}< \epsilon_{i}\leq t_{i}} \bigl\vert T_{\epsilon_{i+1}}f(x)-T_{\epsilon _{i}}f(x) \bigr\vert ^{2}\Biggr)^{1/2}, \end{aligned}$$

where $\{t_{i}\}$ is a fixed sequence which decreases to zero.

Variation and oscillation can be used to measure the speed of convergence of certain convergent families of operators. The operators of variation and oscillation have attracted many researchers’ attention in probability, ergodic theory, and harmonic analysis. Bourgain [1] obtained variation inequality for the ergodic averages of a dynamic system, his work has launched a new research direction in harmonic analysis. Campbell, Jones, Reinhold, and Wierdl in [2] established the $L^{p}$-boundedness of variation operator and oscillation operator for the Hilbert transform. Recently, many other publications have enriched this research direction [3–7].

Let $1\leq r\leq\infty, m\in\mathbb{N}\cup\{0\}$. We say that the kernel *K* satisfies the $H_{r,m}$-Hörmander condition if there exist the constants $c\geq1$ and $C_{r,m}>0$ such that, for all $y\in\mathbb{R}^{n}$ and $R>c \vert x \vert $,

$$\begin{aligned} \sum_{k=1}^{\infty}k^{m} \bigl(2^{k}R\bigr)^{n}\biggl(\frac{1}{(2^{k}R)^{n}} \int_{2^{k}R< \vert y \vert \leq 2^{k+1}R} \bigl\vert K(x-y)-K(-y) \bigr\vert ^{r}\,dy\biggr)^{1/r}\leq C_{r,m}, \end{aligned}$$

if $r<\infty$, and

$$\begin{aligned} \sum_{k=1}^{\infty}k^{m} \bigl(2^{k}R\bigr)^{n}\sup_{2^{k}R< \vert y \vert \leq 2^{k+1}R} \bigl\vert K(x-y)-K(-y) \bigr\vert \leq C_{\infty} \end{aligned}$$

in the case $r=\infty$.

We notice that $H_{r,0}$ is $L^{r}$-Hörmander condition, which was studied in depth in [8].

Let *K* satisfy (1) and $H_{r,1}$-Hörmander condition, and let $T_{b}=\{T_{\epsilon,b}\}_{\epsilon>0}$, where $T_{\epsilon ,b}$ is the commutator of $T_{\epsilon}$ and *b*,

3 $$\begin{aligned} T_{\epsilon,b} f(x)= \int_{ \vert x-y \vert >\epsilon}\bigl(b(x)-b(y)\bigr)K(x-y)f(y)\,dy. \end{aligned}$$

Suppose $r>1,\rho>2$, and $b\in BMO(\mathbb{R}^{n})$. Zhang and Wu proved in [9] that, if $V_{\rho}(T)$ and $\mathcal {O}(T)$ are bounded on $L^{p_{0}}(\mathbb{R}^{n})$ for some $p_{0}>1$, then $V_{\rho}(T_{b})$ and $\mathcal{O}(T_{b}) $ are bounded on $L^{p}(\mathbb{R}^{n}, \omega)$ for any $\max\{r',p_{0}\}< p<\infty, \omega \in A_{p/\max\{r',p_{0}\}}(\mathbb{R}^{n})$.

Given *m* is a positive integer, and *b* is a function on $\mathbb{R}^{n}$. Let $R_{m+1}(b;x,y)$ be the $m+1$th Taylor series remainder of *b* at *x* expander about *y*, *i.e.*,

$$\begin{aligned} R_{m+1}(b;x,y)=b(x)-\sum_{ \vert \alpha \vert \leq m} \frac{1}{\alpha!} D^{\alpha}b(y) (x-y)^{\alpha}. \end{aligned}$$

We consider the family of operators $T^{b}=\{T^{b}_{\epsilon}\}_{\epsilon >0}$, where $T^{b}_{\epsilon}$ are the multilinear singular integral operators of $T_{\epsilon}$ as follows:

4 $$\begin{aligned} T^{b}_{\epsilon}f(x)= \int_{ \vert x-y \vert >\epsilon}\frac{ R_{m+1}(b;x,y)}{ \vert x-y \vert ^{m}}K(x-y)f(y)\,dy. \end{aligned}$$

Note that when $m=0, T^{b}_{\epsilon}$ is just the commutator of $T_{\epsilon}$ and *b*. However, when $m>0, T^{b}_{\epsilon}$ is a non-trivial generation of the commutator. It is well known that multilinear operators have been widely studied by many authors (see [10–14]).

In [15], Hu and Wang established the weighted $(L^{p},L^{q})$ inequalities of the variation and oscillation operators for the multilinear Calderón–Zygmund singular integral with a Lipschitz function in $\mathbb{R}$. In this paper, if *K* satisfies (1) and $H_{r,1}$-Hörmander condition, we will study the bounded behaviors of variation and oscillation operators for the family of the multilinear singular integrals defined by (4) in $L^{p}(\mathbb{R}^{n},\omega(x)\,dx)$ when $D^{\alpha}b\in BMO(\mathbb{R}^{n})$ for $\vert \alpha \vert =m$. Our main results can be formulated as follows.

### Theorem 1

*Let* *K* *satisfy* (1), $\rho>2$, *and let* $T=\{T_{\epsilon}\}_{\epsilon>0}$ *and* $T^{b}=\{T^{b}_{\epsilon}\} _{\epsilon>0}$ *be given by* (2) *and* (4), *respectively*. *Suppose that* $K\in H_{r,1}$,$V_{\rho}(T)$, *and* $\mathcal{O}(T) $ *are bounded on* $L^{p_{0}}(\mathbb{R}^{n})$ *for some* $p_{0}>1$, *and* $D^{\alpha}b\in BMO(\mathbb{R}^{n})$ *for* $\vert \alpha \vert =m$, *then* $V_{\rho}(T^{b})$ *and* $\mathcal{O}(T^{b}) $ *are bounded on* $L^{p}(\mathbb {R}^{n},\omega)$ *for any* $\max\{r',p_{0}\}< p<\infty, \omega\in A_{p/\max\{ r',p_{0}\}}(\mathbb{R}^{n})$.

### Corollary 1

([9])

*Let* *K* *satisfy* (1), $\rho>2$, *and let* $T=\{T_{\epsilon}\} _{\epsilon>0}$ *and* $T_{b}=\{T_{\epsilon,b}\}_{\epsilon>0}$ *be given by* (2) *and* (3), *respectively*. *Suppose that* $K\in H_{r,1}$,$V_{\rho}(T)$ *and* $\mathcal{O}(T) $ *are bounded on* $L^{p_{0}}(\mathbb{R}^{n})$ *for some* $p_{0}>1$, *and* $b\in BMO(\mathbb{R}^{n})$, *then* $V_{\rho}(T_{b})$ *and* $\mathcal{O}(T_{b}) $ *are bounded on* $L^{p}(\mathbb{R}^{n},\omega)$ *for any* $\max\{r',p_{0}\} < p<\infty, \omega\in A_{p/\max\{r',p_{0}\}}(\mathbb{R}^{n})$.

In this paper, we shall use the symbol $A\lesssim B$ to indicate that there exists a universal positive constant *C*, independent of all important parameters, such that $A\leq CB$. $A\thickapprox B$ means that $A\lesssim B$ and $B\lesssim A$.

## Some preliminaries

### $A_{p}(\mathbb{R}^{n})$ weight

A weight *ω* belongs to $A_{p}(\mathbb{R}^{n})$ for $1< p<\infty$ if there exists a constant *C* such that, for every ball $B\subset\mathbb{R}^{n} $,

$$\begin{aligned} \biggl(\frac{1}{ \vert B \vert } \int_{B}\omega(x)\,dx\biggr) \biggl(\frac{1}{ \vert B \vert } \int_{B}\omega(x)^{1-p^{\prime}}\,dx\biggr)^{p-1}\leq C, \end{aligned}$$

where $p^{\prime}$ is the dual of *p* such that $1/p+1/p^{\prime}=1$. A weight *ω* belongs to $A_{1}(\mathbb{R}^{n})$ if

$$\begin{aligned} \frac{1}{ \vert B \vert } \int_{B}w(y)\,dy\leq C\cdot\mathop{\operatorname{ess\, inf}}_{x\in B} w(x) \quad\mbox{for every ball } B\subset\mathbb{R}^{n}. \end{aligned}$$

### Function of $BMO(\mathbb{R}^{n})$

Following [16], a locally integrable function *b* is said to be in $BMO(\mathbb{R}^{n})$ if

$$\sup_{B\subset\mathbb{R}^{n} }\frac{1}{ \vert B \vert } \int_{B} \bigl\vert b(x)-b_{B} \bigr\vert \,dx= \Vert b \Vert _{*}< \infty, $$

where

$$b_{B}=\frac{1}{ \vert B \vert } \int_{B} b(y)\,dy. $$

The function of $BMO(\mathbb{R}^{n})$ has the following property.

#### Lemma 1

*Suppose* $b\in BMO(\mathbb{R}^{n}),B_{k}=2^{k}B, k\in\mathbb{N}\cup\{0\}$. *Then*, *for* $1\leq p<\infty$,

$$\begin{aligned} \biggl(\frac{1}{ \vert B \vert } \int_{B} \bigl\vert b(x)-b_{B_{k}} \bigr\vert ^{p}\,dx\biggr)^{1/p}\lesssim k \Vert b \Vert _{*}. \end{aligned}$$

### Maximal function

The Hardy–Littlewood maximal operator is defined by

$$\begin{aligned} M(f) (x)=\sup_{B\ni x}\frac{1}{ \vert B \vert } \int_{B} \bigl\vert f(y) \bigr\vert \,dy. \end{aligned}$$

We also define the maximal function

$$\begin{aligned} M_{r}(f) (x)=\sup_{B\ni x}\biggl(\frac{1}{ \vert B \vert } \int_{B} \bigl\vert f(y) \bigr\vert ^{r}\,dy \biggr)^{1/r} \end{aligned}$$

for $1\leq r<\infty$. It is well known that $M_{r}$ is bounded on $L^{p}(\mathbb{R}^{n},\omega)$ for $r< p<\infty$ and $\omega\in A_{p}(\mathbb{R}^{n})$ (see [17]).

### Taylor series remainder

By definition, it is obvious that

$$\begin{aligned} R_{m+1}(b;x,y)=R_{m}(b;x,y)-\sum _{ \vert \alpha \vert =m}\frac{1}{\alpha!}D^{\alpha}b(y) (x-y)^{\alpha}. \end{aligned}$$

The following lemma gives an estimate on Taylor series remainder.

#### Lemma 2

([11])

*Let* *b* *be a function on* $\mathbb{R}^{n}$ *with* *mth order derivatives in* $L^{q}(\mathbb{R})$ *for some* $q>n$. *Then*

$$\begin{aligned} \bigl\vert R_{m}(b;x,y) \bigr\vert \lesssim \vert x-y \vert ^{m}\sum_{ \vert \alpha \vert =m}\biggl(\frac {1}{ \vert Q(x,y) \vert } \int_{Q(x,y)} \bigl\vert D^{\alpha}b(z) \bigr\vert ^{q}\,dz\biggr)^{1/q}, \end{aligned}$$

*where* $Q(x,y)$ *is the cube centered at* *x* *and having diameter* $5\sqrt{n} \vert x-y \vert $.

### Variation and oscillation operators

Following [2], let $\Theta=\{\beta: \beta=\{\epsilon_{i}\},\epsilon _{i}\in\mathbb{R},\epsilon_{i}\searrow0\}$. We denote by $F_{\rho}$ the mixed norm space of two variable functions $g(i,\beta)$ such that

$$\begin{aligned} \Vert g \Vert _{F_{\rho}}\equiv\sup_{\beta}\biggl(\sum _{i} \bigl\vert g(i,\beta) \bigr\vert ^{\rho}\biggr)^{1/\rho}. \end{aligned}$$

We also consider the $F_{\rho}$-valued operator $\mathcal {V}(T):f\rightarrow\mathcal{V}(T)f$ by

$$\begin{aligned} \mathcal{V}(T)f(x)=\bigl\{ T_{\epsilon_{i+1}}f(x)-T_{\epsilon_{i}}f(x)\bigr\} _{\beta=\{\epsilon_{i}\}\in\Theta}. \end{aligned}$$

This implies that

$$\begin{aligned} \mathcal{V}_{\rho}(T)f(x)= \bigl\Vert \mathcal{V}(T)f(x) \bigr\Vert _{F_{\rho}}. \end{aligned}$$

On the other hand, we consider the operator

$$\begin{aligned} \mathcal{O}'(Tf) (x)=\Biggl(\sum_{i=1}^{\infty}\sup_{t_{i+1}< \delta_{i}< t_{i}} \bigl\vert T_{t_{i+1}}f(x)-T_{\delta_{i}}f(x) \bigr\vert ^{2}\Biggr)^{1/2}. \end{aligned}$$

It is easy to check that

$$\begin{aligned} \mathcal{O}'(Tf)\thickapprox\mathcal{O}(Tf). \end{aligned}$$

We denote by *E* the mixed norm Banach space of two-variable function *h* defined on $\mathbb{R}\times\mathbb{N}$ such that

$$\begin{aligned} \Vert h \Vert _{E}\equiv\biggl(\sum_{i} \Bigl(\sup_{s} \bigl\vert h(s,i) \bigr\vert \Bigr)^{2} \biggr)^{1/2}< \infty. \end{aligned}$$

For a fixed decreasing sequence $\{t_{i}\}$ with $t_{i}\searrow0$, let $J_{i}=(t_{i+1},t_{i}]$ and define the *E*-valued operator $\mathcal{U}(T): f\rightarrow\mathcal{U}(T)f$ given by

$$\begin{aligned} \mathcal{U}(T)f(x)=\bigl\{ T_{t_{i+1}}f(x)-T_{s}f(x)\bigr\} _{s\in J_{i},i\in \mathbb{N}}=\biggl\{ \int_{\{t_{i+1}< \vert x-y \vert < s\}}K(x,y)f(y)\,dy\biggr\} _{s\in J_{i},i\in\mathbb{N}}. \end{aligned}$$

Then

$$\begin{aligned} \mathcal{O}'(Tf) (x)={}&\bigl\Vert \mathcal{U}(T)f(x) \bigr\Vert _{E}= \bigl\Vert \bigl\{ T_{t_{i+1}}f(x)-T_{s}f(x) \bigr\} _{s\in J_{i},i\in\mathbb{N}} \bigr\Vert _{E} \\ ={}&\biggl\Vert \biggl\{ \int_{\{t_{i+1}< \vert x-y \vert < s\}}K(x,y)f(y)\,dy\biggr\} _{s\in J_{i},i\in\mathbb{N}} \biggr\Vert _{E}. \end{aligned}$$

Let *B* be a Banach space and *φ* be a *B*-valued function, we define the sharp maximal operator as follows:

$$\begin{aligned} \varphi^{\sharp}(x)=\sup_{x\in I}\frac{1}{ \vert I \vert } \int_{I} \biggl\Vert \varphi (y)-\frac{1}{ \vert I \vert } \int_{I}\varphi(z)\,dz \biggr\Vert _{B}\,dy. \end{aligned}$$

Then

$$\begin{aligned} M^{\sharp}\bigl(\mathcal{\mathcal{V}}_{\rho}(Tf)\bigr)\leq2\bigl( \mathcal{V}(T)f\bigr)^{\sharp}(x) \end{aligned}$$

and

$$\begin{aligned} M^{\sharp}\bigl(\mathcal{O}'(Tf)\bigr)\leq2\bigl( \mathcal{U}(T)f\bigr)^{\sharp}(x). \end{aligned}$$

Finally, let us recall some results about the boundedness of $V_{\rho}(T)$ and $\mathcal{O}(T) $.

#### Lemma 3

([9])

*Let* *K* *satisfy* (1), $\rho>2$, $T=\{T_{\epsilon}\} _{\epsilon>0}$ *be given by* (2). *Suppose that* *K* *satisfies* (1) *and* $K\in H_{r,0}$-*Hörmander condition*, $V_{\rho}(T)$ *and* $\mathcal{O}(T) $ *are bounded on* $L^{p_{0}}(\mathbb{R}^{n})$ *for some* $1< p_{0}<\infty$. *Then* $V_{\rho}(T)$ *and* $\mathcal{O}(T) $ *are bounded on* $L^{p}(\mathbb {R}^{n})$ *for any* $\max\{r',p_{0}\}< p<\infty$.

## Proof of Theorem 1

By the fact that $M_{s}$ is bounded on $L^{p}(\mathbb{R}^{n},\omega)$ for $1\leq s< p<\infty$ and $\omega\in A_{p}(\mathbb{R}^{n})$, we need to prove

5 $$\begin{aligned} M^{\sharp}(\mathcal{V}_{\rho}\bigl(T^{b} \bigr)f(x) \lesssim\sum_{ \vert \alpha \vert =m} \bigl\Vert D^{\alpha}{b} \bigr\Vert _{*} M_{s}(f) (x) \end{aligned}$$

and

6 $$\begin{aligned} M^{\sharp}(\mathcal{O}'\bigl(T^{b} \bigr)f(x) \lesssim\sum_{ \vert \alpha \vert =m} \bigl\Vert D^{\alpha}{b} \bigr\Vert _{*} M_{s}(f) (x) \end{aligned}$$

for any $s> \max\{r',p_{0}\}$.

We will only need to prove (5), since (6) can be obtained by a similar argument. To prove (5), it suffices to prove that for any fixed $x_{0}\in\mathbb{R}^{n}$ and some ${F_{\rho}}$-valued constant $c_{1}$, such that for every ball $B=B(x_{0},l)$ with radius *l*, centered at $x_{0}$, the following inequality

$$\begin{aligned} \frac{1}{ \vert B \vert } \int_{B} \bigl\Vert V\bigl(T^{b} \bigr)f(x)-c_{1} \bigr\Vert _{E}\,dx \lesssim\sum _{ \vert \alpha \vert =m} \bigl\Vert D^{\alpha}{b} \bigr\Vert _{*} M_{s}(f) (x_{0}) \end{aligned}$$

holds.

We write $f=f_{1}+f_{2}=f\chi_{10B}+f\chi_{\mathbb{R}^{n}\setminus10B}$. Then

$$\begin{aligned} V\bigl(T^{b}\bigr)f(x) ={}&\biggl\{ \int_{\{\epsilon_{i+1}< \vert x-y \vert < \epsilon_{i}\}}\frac {R_{m+1}({b};x,y)}{ \vert x-y \vert ^{m}}K(x-y)f(y)\,dy\biggr\} _{\beta=\{\epsilon_{i}\} \in\Theta} \\ ={}&V(T) \biggl(\frac{R_{m+1}({b};x,\cdot)}{ \vert x-\cdot \vert ^{m}}f_{1} \biggr) (x)+V \bigl(T^{b}\bigr) (f_{2}) (x). \end{aligned}$$

Let $x_{1}$ be a point at the boundary of 2*B*, and let

$$\begin{aligned} c_{1}=\biggl\{ \int_{\{\epsilon_{i+1}< \vert x_{1}-y \vert < \epsilon_{i}\}}\frac {R_{m+1}({b};x_{1},y)}{ \vert x_{1}-y \vert ^{m}}K(x_{1}-y)f_{2}(y)\,dy \biggr\} _{\beta=\{ \epsilon_{i}\}\in\Theta}=V(T) (f_{2}) (x_{1}). \end{aligned}$$

Then

$$\begin{aligned} &\frac{1}{ \vert B \vert } \int_{B} \bigl\Vert V\bigl(T^{b} \bigr)f(x)-c_{1} \bigr\Vert _{F_{\rho}}\,dx \\ &\quad\leq\frac{1}{ \vert B \vert } \int_{B} \biggl\Vert V(T) \biggl(\frac{R_{m+1}({b};x,\cdot )}{ \vert x-\cdot \vert ^{m}}f_{1} \biggr) (x) \biggr\Vert _{F_{\rho}}\,dx \\ &\qquad{}+\frac{1}{ \vert B \vert } \int_{B} \bigl\Vert V\bigl(T^{b}\bigr) (f_{2}) (x)-V\bigl(T^{b}\bigr) (f_{2}) (x_{1}) \bigr\Vert _{F_{\rho}}\,dx \\ &\quad=M_{1}+M_{2}. \end{aligned}$$

For any $x\in B, k\in\mathbb{Z}$, let $E_{k}=\{y:2^{k}\cdot3l\leq \vert y-x \vert <2^{k+1}\cdot3l\}$, let $F_{k}=\{y: \vert y-x \vert <2^{k+1}\cdot3l\}$, and let

$$\begin{aligned} {b}_{k}(z)=b(z)-\sum_{ \vert \alpha \vert =m} \frac{1}{\alpha!}\bigl(D^{\alpha}b\bigr)_{F_{k}}z^{m}. \end{aligned}$$

By [11] we have $R_{m+1}({b};x,y)=R_{m+1}(b_{k};x,y)$ for any $y\in E_{k}$. From Lemma 3, we know $V_{\rho}(T)$ is bounded on $L^{u}(\mathbb{R}^{n})$ for $\max\{r',p_{0}\}< u< s$. Then, using Hölder’s inequality, we deduce

$$\begin{aligned} M_{1}\lesssim{}& \biggl(\frac{1}{ \vert B \vert } \int_{B} \biggl\Vert V(T) \biggl(\frac{R_{m+1}({b};x,\cdot )}{ \vert x-\cdot \vert ^{m}}f_{1} \biggr) \biggr\Vert ^{u}_{F_{\rho}}\,dx\biggr)^{1/u} \\ \lesssim{}& \biggl(\frac{1}{ \vert B \vert } \int_{\{y: \vert y-x \vert < 11l\}}\biggl\vert \frac{R_{m+1}({b};\cdot ,y)}{ \vert y-\cdot \vert ^{m}}f(y) \biggr\vert ^{u}\,dy \biggr)^{1/u} \\ ={}& \Biggl(\frac{1}{ \vert B \vert } \int_{\{y: \vert y-x \vert < 11l\}} \Biggl\vert \sum_{k=-\infty }^{1} \biggl(\frac{R_{m+1}({b}_{k};\cdot,y)}{ \vert y-\cdot \vert ^{m}}\chi_{E_{k}}(y) \biggr)f(y) \Biggr\vert ^{u}\,dy\Biggr)^{1/u} \\ \lesssim{}& \Biggl(\frac{1}{ \vert B \vert } \int_{\{y: \vert y-x \vert < 11l\}} \Biggl\vert \Biggl(\sum_{k=-\infty }^{1} \frac{R_{m}({b_{k}};\cdot,y)}{ \vert y-\cdot \vert ^{m}}\chi_{E_{k}}(y) \\ &{}-\frac{1}{ \vert y-\cdot \vert ^{m}}\sum_{ \vert \alpha \vert =m}\frac{1}{\alpha!} \sum_{k=-\infty}^{1}D^{\alpha}b_{k}(y)\chi_{E_{k}}(y) (y-\cdot)^{\alpha}\Biggr)f(y) \Biggr\vert ^{u}\,dy\Biggr)^{1/u} \\ \lesssim{}& \Biggl(\frac{1}{ \vert B \vert }\sum_{k=-\infty}^{1} \int_{E_{k}}\biggl\vert \frac {R_{m}({b_{k}};\cdot,y)}{ \vert y-\cdot \vert ^{m}}f(y) \biggr\vert ^{u}\,dy \Biggr)^{1/u} \\ &{}+\sum_{ \vert \alpha \vert =m}\Biggl(\frac{1}{ \vert B \vert } \int_{\{y: \vert y-x \vert < 11l\}} \Biggl(\Biggl(\sum_{k=-\infty}^{1} \bigl\vert D^{\alpha}b_{k}(y) \bigr\vert \chi_{E_{k}}(y)\Biggr) \bigl\vert f(y)\bigr\vert \Biggr)^{u}\,dy\Biggr)^{1/u} \\ = {}&M_{11}+M_{12}. \end{aligned}$$

For any $y\in E_{k}$, by Lemma 2 and Lemma 1,

$$\begin{aligned} \bigl\vert R_{m}({b}_{k};x,y) \bigr\vert & \lesssim \vert x-y \vert ^{m}\sum_{ \vert \alpha \vert =m}\biggl( \frac {1}{ \vert Q(x,y) \vert } \int_{Q(x,y)} \bigl\vert D^{\alpha}b(z) \bigr\vert ^{q}\,dz\biggr)^{1/q} \\ &\lesssim \vert x-y \vert ^{m}\sum_{ \vert \alpha \vert =m} \biggl(\frac{1}{(\sqrt{n}2^{k}\cdot 30l)^{n}} \int_{ \vert z-x \vert < \sqrt{n}2^{k}\cdot30l} \bigl\vert D^{\alpha}b(z)- \bigl(D^{\alpha}b\bigr)_{F_{k}} \bigr\vert ^{q}\,dz \biggr)^{1/q} \\ &\lesssim \vert x-y \vert ^{m}\sum_{ \vert \alpha \vert =m} \bigl\Vert D^{\alpha}{b} \bigr\Vert _{*}. \end{aligned}$$

Then we have

$$\begin{aligned} M_{11} \lesssim{}&\sum_{ \vert \alpha \vert =m} \bigl\Vert D^{\alpha}{b} \bigr\Vert _{*}\Biggl(\frac {1}{ \vert B \vert }\sum _{k=-\infty}^{1} \int_{E_{k}} \bigl\vert f(y) \bigr\vert ^{u}\,dy \Biggr)^{1/u} \\ = {}&\sum_{ \vert \alpha \vert =m} \bigl\Vert D^{\alpha}{b} \bigr\Vert _{*}\biggl(\frac{1}{ \vert B \vert } \int _{12B} \bigl\vert f(y) \bigr\vert ^{u}\,dy \biggr)^{1/u} \\ \lesssim{}& \sum_{ \vert \alpha \vert =m} \bigl\Vert D^{\alpha}{b} \bigr\Vert _{*} M_{s}(f) (x_{0}). \end{aligned}$$

By $D^{\alpha}b_{k}(y)=D^{\alpha}b(y)-(D^{\alpha}b)_{F_{k}}$, applying Hölder’s inequality and Lemma 2, we get

$$\begin{aligned} M_{12} \lesssim{}&\Biggl(\frac{1}{ \vert B \vert }\biggl( \int_{12B} \bigl\vert f(y) \bigr\vert ^{s}\,dy \biggr)^{u/s}\Biggl(\sum_{ \vert \alpha \vert =m}\sum _{k=-\infty}^{1} \int_{F_{k}} \bigl\vert D^{\alpha}b(y)- \bigl(D^{\alpha}b\bigr)_{F_{k}} \bigr\vert ^{\frac{us}{s-u}}\,dy \Biggr)^{1-u/s} \Biggr)^{1/u} \\ \lesssim{}&\sum_{ \vert \alpha \vert =m} \bigl\Vert D^{\alpha}{b} \bigr\Vert _{*} \Biggl(\frac{1}{ \vert B \vert } \biggl( \int_{12B} \bigl\vert f(y) \bigr\vert ^{s}\,dy \biggr)^{u/s} \sum_{k=-\infty}^{1} \vert F_{k} \vert ^{1-u/s}\Biggr)^{1/u} \\ \lesssim{}&\sum_{ \vert \alpha \vert =m} \bigl\Vert D^{\alpha}{b} \bigr\Vert _{*}\biggl(\frac{1}{ \vert B \vert } \int _{12B} \bigl\vert f(y) \bigr\vert ^{s}\,dy \biggr)^{1/s} \lesssim\sum_{ \vert \alpha \vert =m} \bigl\Vert D^{\alpha}{b} \bigr\Vert _{*} M_{s}(f) (x_{0}). \end{aligned}$$

We now estimate $M_{2}$. We write

$$\begin{aligned} &\bigl\Vert V\bigl(T^{b}\bigr)f_{2}(x)-V \bigl(T^{b}\bigr)f_{2}(x_{1}) \bigr\Vert _{F_{\rho}} \\ &\quad= \biggl\Vert \biggl\{ \int_{\{\epsilon_{i+1}< \vert x-y \vert < \epsilon_{i}\}}\frac {R_{m+1}({b};x,y)}{ \vert x-y \vert ^{m}}K(x-y)f_{2}(y)\,dy\\ &\qquad{} - \int_{\{\epsilon_{i+1}< \vert x_{1}-y \vert < \epsilon_{i}\}}\frac {R_{m+1}({b};x_{1},y)}{ \vert x_{1}-y \vert ^{m}}K(x_{1}-y)f_{2}(y)\,dy \biggr\} _{\beta=\{ \epsilon_{i}\}\in\Theta} \biggr\Vert _{F_{\rho}}\\ &\quad \leq \biggl\Vert \biggl\{ \int_{\{\epsilon_{i+1}< \vert x-y \vert < \epsilon_{i}\}} \biggl(\frac{R_{m+1}({b};x,y)}{ \vert x-y \vert ^{m}}K(x-y)\\ &\qquad{} - \frac{R_{m+1}({b};x_{1},y)}{ \vert x_{1}-y \vert ^{m}}K(x_{1}-y) \biggr)f_{2}(y)\,dy\biggr\} _{\beta=\{\epsilon_{i}\}\in\Theta} \biggr\Vert _{F_{\rho}}\\ &\qquad{} + \biggl\Vert \biggl\{ \int_{\mathbb{R}^{n}}\bigl(\chi_{\{\epsilon _{i+1}< \vert x-y \vert < \epsilon_{i}\}}(y)-\chi_{\{\epsilon_{i+1}< \vert x_{1}-y \vert < \epsilon _{i}\}}(y) \bigr) \\ &\qquad{}\times\frac {R_{m+1}({b};x_{1},y)}{ \vert x_{1}-y \vert ^{m}}K(x_{1}-y)f_{2}(y)\,dy \biggr\} _{\beta=\{\epsilon _{i}\}\in\Theta} \biggr\Vert _{F_{\rho}} \\ &\quad = N_{1}+N_{2}. \end{aligned}$$

By Minkowski’s inequality and $\Vert \{\epsilon _{i+1}< \vert x-y \vert <\epsilon_{i}\}_{\beta=\{\epsilon_{i}\}\in\Theta} \Vert _{F_{\rho}}\leq1$, we obtain

$$\begin{aligned} N_{1}\leq{}&\int_{\mathbb{R}^{n}} \bigl\Vert \{\chi_{\{t_{i+1}< \vert x-y \vert < s\}} \}_{\beta=\{\epsilon_{i}\}\in\Theta} \bigr\Vert _{F_{\rho}} \\ &{}\times \biggl\vert \frac{R_{m+1}({b};x,y)}{ \vert x-y \vert ^{m}}K(x-y) -\frac{R_{m+1}({b};x_{1},y)}{ \vert x_{1}-y \vert ^{m}}K(x_{1}-y) \biggr\vert \bigl\vert f_{2}(y) \bigr\vert \,dy \\ \leq{}&\int_{\mathbb{R}^{n}} \biggl\vert \frac{R_{m+1}({b};x,y)}{ \vert x-y \vert ^{m}}K(x-y) - \frac{R_{m+1}({b};x_{1},y)}{ \vert x_{1}-y \vert ^{m}}K(x_{1}-y) \biggr\vert \bigl\vert f_{2}(y) \bigr\vert \,dy \\ \leq{}&\sum_{k=1}^{\infty}\int_{E_{k}} \biggl\vert \biggl(\frac {R_{m+1}({b}_{k};x,y)}{ \vert x-y \vert ^{m}}- \frac {R_{m+1}({b}_{k};x_{1},y)}{ \vert x_{1}-y \vert ^{m}}\biggr)K(x-y) \biggr\vert \bigl\vert f_{2}(y) \bigr\vert \,dy \\ &{}+ \int_{\mathbb{R}^{n}} \biggl\vert \frac{R_{m+1}({b};x_{1},y)}{ \vert x_{1}-y \vert ^{m}} \biggr\vert \bigl\vert K(x-y)-K(x_{1}-y) \bigr\vert \bigl\vert f_{2}(y) \bigr\vert \,dy \\ \leq{}& \sum_{k=1}^{\infty}\int_{E_{k}}\frac {1}{ \vert x-y \vert ^{m}}\vert R_{m}({b}_{k};x,y)-R_{m}({b}_{k};x_{1},y) \bigl\vert K(x-y) \bigr\vert \bigl\vert f(y) \bigr\vert \,dy \\ &{}+\sum _{k=1}^{\infty}\int_{E_{k}} \bigl\vert R_{m}({b}_{k};x_{1},y) \bigr\vert \biggl\vert \frac {1}{ \vert x-y \vert ^{m}}-\frac{1}{ \vert x_{1}-y \vert ^{m}} \biggl\vert \bigl\vert K(x-y) \bigr\vert \bigl\vert f(y) \bigr\vert \,dy\\ &{} +\sum _{k=1}^{\infty}\int_{E_{k}}\sum_{ \vert \alpha \vert =m} \frac{1}{\alpha !} \bigr\vert D^{\alpha}{b}_{k}(y) \bigr\vert \biggl\vert \frac{(x-y)^{\alpha}}{ \vert x-y \vert ^{m}}- \frac{(x_{1}-y)^{\alpha}}{ \vert x_{1}-y \vert ^{m}} \biggr\vert \bigl\vert K(x-y) \bigr\vert \bigl\vert f(y) \bigr\vert \,dy\\ &{} + \int_{(10B)^{c}} \biggl\vert \frac{R_{m+1}({b};x_{1},y)}{ \vert x_{1}-y \vert ^{m}} \biggr\vert \bigl\vert K(x-y)-K(x_{1}-y) \bigr\vert \bigl\vert f(y) \bigr\vert \,dy\\ ={}& N_{11}+N_{12}+N_{13}+N_{14}. \end{aligned}$$

Applying the formula ([11] p. 448), we have

$$\begin{aligned} R_{m}({b}_{k};x,y)-R_{m}({b}_{k};x_{1},y) =R_{m}({b}_{k};x,x_{1})+\sum _{0< \vert \beta \vert < m}\frac{(x-x_{1})^{\beta}}{\beta !}R_{m-\beta}\bigl(D^{\beta}{b}_{k};x_{1},y \bigr). \end{aligned}$$

Then, by Lemmas 1 and 2, we have

$$\begin{aligned} \bigl\vert R_{m}({b}_{k};x,x_{1}) \bigr\vert \lesssim{}&\vert x-x_{1} \vert ^{m}\sum _{ \vert \alpha \vert =m}\biggl(\frac {1}{Q(x,x_{1})} \int_{Q(x,x_{1})} \bigl\vert D^{\alpha}b_{k}- \bigl(D^{\alpha}b_{k}\bigr)_{F_{k}} \bigr\vert ^{q} \biggr)^{1/q} \\ \lesssim{}&kl^{m}\sum_{ \vert \alpha \vert =m} \bigl\Vert D^{\alpha}b \bigr\Vert _{*} \end{aligned}$$

and

$$\begin{aligned} \bigl\vert R_{m-\beta}\bigl(D^{\beta}{b}_{k};x_{1},y \bigr) \bigr\vert \lesssim \vert x-y \vert ^{m-\beta}\sum _{ \vert \alpha \vert =m} \bigl\Vert D^{\alpha}b \bigr\Vert _{*}. \end{aligned}$$

So

$$\begin{aligned} \bigl\vert R_{m}({b}_{k};x,y)-R_{m}({b}_{k};x_{1},y) \bigr\vert \lesssim{}& \sum_{ \vert \alpha \vert =m} \bigl\Vert D^{\alpha}b \bigr\Vert _{*}\biggl(kl^{m}+\sum _{0< \vert \beta \vert < m}l^{\beta} \vert x-y \vert ^{m-\beta} \biggr) \\ \lesssim{}&kl \vert x-y \vert ^{m-1}\sum _{ \vert \alpha \vert =m} \bigl\Vert D^{\alpha}b \bigr\Vert _{*}. \end{aligned}$$

By (1) we have

$$\begin{aligned} \bigl\vert K(x-y) \bigr\vert \lesssim \vert x-y \vert ^{-n}. \end{aligned}$$

Then

$$\begin{aligned} N_{11}={}& \sum_{k=1}^{\infty}\int_{E_{k}}\frac {1}{ \vert x-y \vert ^{m}} \bigl\vert R_{m}({b}_{k};x,y)-R_{m}({b}_{k};x_{1},y) \bigr\vert K(x-y)\vert \bigl\vert f(y) \bigr\vert \,dy \\ \lesssim{}& \sum _{ \vert \alpha \vert =m} \bigl\Vert D^{\alpha}b \bigr\Vert _{*} \sum_{k=1}^{\infty}\int _{E_{k}}\frac{kl}{ \vert x-y \vert ^{n+1}} \bigl\vert f(y) \bigr\vert \,dy\\ \lesssim{}& \sum_{ \vert \alpha \vert =m} \bigl\Vert D^{\alpha}b \bigr\Vert _{*}\sum_{k=1}^{\infty}\frac {k}{2^{k}}\frac{1}{ \vert F_{k} \vert } \int_{F_{k}} \bigl\vert f(y) \bigr\vert \,dy\\ \lesssim{}& \sum _{ \vert \alpha \vert =m} \bigl\Vert D^{\alpha}b \bigr\Vert _{*}M_{s}(f) (x_{0}). \end{aligned}$$

For $y\in E_{k}, x\in B$ and $x_{1}$ being a point at the boundary of 2*B*, we get $\vert x_{1}-y \vert \thickapprox \vert x-y \vert $. Thus

$$\begin{aligned} \biggl\vert \frac{1}{ \vert x-y \vert ^{m}}-\frac{1}{ \vert x_{1}-y \vert ^{m}} \biggr\vert \lesssim \frac { \vert x-x_{1} \vert }{ \vert x-y \vert ^{m+1}}\lesssim\frac{l}{ \vert x-y \vert ^{m+1}} \end{aligned}$$

and

$$\begin{aligned} \bigl\vert R_{m}({b}_{k};x_{1},y) \bigr\vert \lesssim \vert x-y \vert ^{m}\sum_{ \vert \alpha \vert =m} \bigl\Vert D^{\alpha}b \bigr\Vert _{*}. \end{aligned}$$

Then

$$\begin{aligned} N_{12}\lesssim \sum_{ \vert \alpha \vert =m} \bigl\Vert D^{\alpha}b \bigr\Vert _{*}\sum_{k=1}^{\infty}\int _{E_{k}}\frac{l \vert f(y) \vert }{ \vert x-y \vert ^{n+1}}\,dy \lesssim \sum_{ \vert \alpha \vert =m} \bigl\Vert D^{\alpha}b \bigr\Vert _{*}M_{s}(f) (x_{0}). \end{aligned}$$

As for $N_{13}$, due to

$$\begin{aligned} \biggl\vert \frac{(x-y)^{\alpha}}{ \vert x-y \vert ^{m}}- \frac{(x_{1}-y)^{\alpha}}{ \vert x_{1}-y \vert ^{m}} \biggr\vert \lesssim \frac{ \vert x-x_{1} \vert }{ \vert x-y \vert } \end{aligned}$$

for $\vert \alpha \vert =m$, and $D^{\alpha}{b}_{k}(y)=D^{\alpha}{b}(y)-(D^{\alpha}{b})_{F_{k}}$, we have

$$\begin{aligned} N_{13}\lesssim{}& \sum_{k=1}^{\infty}\int_{E_{k}}\frac {l \vert f(y) \vert }{ \vert x-y \vert ^{n+1}}\sum_{ \vert \alpha \vert =m} \bigl\vert D^{\alpha}b(y)-\bigl(D^{\alpha}b\bigr)_{F_{k}} \bigr\vert \,dy \\ \lesssim{}&\sum_{k=1}^{\infty}\frac{1}{2^{k}} \frac{1}{ \vert F_{k} \vert } \int _{F_{k}} \bigl\vert f(y) \bigr\vert \sum _{ \vert \alpha \vert =m} \bigl\vert D^{\alpha}b(y)-\bigl(D^{\alpha}b\bigr)_{F_{k}} \bigr\vert \,dy \\ \lesssim{}& \sum_{k=1}^{\infty}\frac{1}{2^{k}} \biggl(\frac{1}{ \vert F_{k} \vert } \int _{F_{k}} \bigl\vert f(y) \bigr\vert ^{s}\,dy \biggr)^{1/s} \sum_{ \vert \alpha \vert =m}\biggl( \frac{1}{ \vert F_{k} \vert } \int_{F_{k}} \bigl\vert D^{\alpha}b(y)- \bigl(D^{\alpha}b\bigr)_{F_{k}} \bigr\vert ^{s'}\,dy \biggr)^{1/s'} \\ \lesssim{}&\sum_{ \vert \alpha \vert =m} \bigl\Vert D^{\alpha}b \bigr\Vert _{*}M_{s}(f) (x_{0})\sum _{k=1}^{\infty}\frac{1}{2^{k}} \\ \lesssim{}& \sum_{ \vert \alpha \vert =m} \bigl\Vert D^{\alpha}b \bigr\Vert _{*}M_{s}(f) (x_{0}). \end{aligned}$$

Let us estimate $N_{14}$ now. For $y\in(10B)^{c}$, we have $\vert y-x_{1} \vert \geq \vert y-x_{0} \vert - \vert x_{0}-x_{1} \vert >8l$. For $k=1,2,\ldots$ , let $\widetilde{E}_{k}=\{y:2^{k}\cdot 3l\leq \vert y-x_{1} \vert <2^{k+1}\cdot3l\}$, let $\widetilde{F}_{k}=\{y: \vert y-x_{1} \vert <2^{k+1}\cdot3l\}$, and let

$$\begin{aligned} {\widetilde{b}}_{k}(z)=b(z)-\sum_{ \vert \alpha \vert =m} \frac{1}{\alpha!}\bigl(D^{\alpha}b\bigr)_{\widetilde{F}_{k}}z^{m}. \end{aligned}$$

Note that $Q(x,y)\subset2\sqrt{n}Q(x_{1},y)$, then for $x\in B, y\in \widetilde{E}_{k}$ we have

$$\begin{aligned} \bigl\vert R_{m}({\widetilde{b}}_{k};x,y) \bigr\vert \lesssim{}& \vert x-y \vert ^{m}\sum_{ \vert \alpha \vert =m} \biggl(\frac{1}{ \vert 2\sqrt{n}Q(x_{1},y) \vert } \int_{2\sqrt{n}Q(x_{1},y)} \bigl\vert D^{\alpha}{\widetilde{b}}_{k}(z) \bigr\vert ^{q}\,dz\biggr)^{1/q} \\ \lesssim{}& \vert x_{1}-y \vert ^{m}\sum _{ \vert \alpha \vert =m} \bigl\Vert D^{\alpha}{b} \bigr\Vert _{*}. \end{aligned}$$

So

$$\begin{aligned} \bigl\vert R_{m+1}({\widetilde{b}}_{k};x,y) \bigr\vert \lesssim \vert x_{1}-y \vert ^{m}\biggl(\sum _{ \vert \alpha \vert =m} \bigl\Vert D^{\alpha}b \bigr\Vert _{*}+\sum _{ \vert \alpha \vert =m} \bigl\vert D^{\alpha}b(y)- \bigl(D^{\alpha}b\bigr)_{\widetilde{F}_{k}} \bigr\vert \biggr). \end{aligned}$$

Then

$$\begin{aligned} N_{14} \lesssim{}& \sum_{ \vert \alpha \vert =m} \bigl\Vert D^{\alpha}b \bigr\Vert _{*}\sum_{k=1}^{\infty}\int _{\widetilde{E}_{k}} \bigl\vert K(x-y)-K(x_{1}-y) \bigr\vert \bigl\vert f(y) \bigr\vert \,dy \\ & {}+\sum_{k=1}^{\infty}\sum _{ \vert \alpha \vert =m} \int_{\widetilde{E}_{k}} \bigl\vert D^{\alpha}b(y)- \bigl(D^{\alpha}b\bigr)_{\widetilde{F}_{k}} \bigr\vert \bigl\vert K(x-y)-K(x_{1}-y) \bigr\vert \bigl\vert f(y) \bigr\vert \,dy \\ ={}& N_{141}+N_{142}. \end{aligned}$$

Taking $R=3l$, then $\vert x-x_{1} \vert < R$. By Hölder’s inequality and $K\in H_{r,1}\subset H_{r,0} $, we have

$$\begin{aligned} N_{141}\lesssim{}& \sum_{ \vert \alpha \vert =m} \bigl\Vert D^{\alpha}b \bigr\Vert _{*}\sum_{k=1}^{\infty}\biggl( \int_{\widetilde{E}_{k}} \bigl\vert K(x-y)-K(x_{1}-y) \bigr\vert ^{r}\,dy \biggr)^{1/r}\biggl( \int_{\widetilde{E}_{k}} \bigl\vert f(y) \bigr\vert ^{r'}\,dy \biggr)^{1/r'} \\ \lesssim{}& \sum_{ \vert \alpha \vert =m} \bigl\Vert D^{\alpha}b \bigr\Vert _{*}\sum_{k=1}^{\infty}\bigl(2^{k} \cdot3l\bigr)^{n}\biggl(\frac{1}{(2^{k}\cdot3l)^{n}} \int_{\widetilde{E}_{k}} \bigl\vert K(x-y)-K(x_{1}-y) \bigr\vert ^{r}\,dy\biggr)^{1/r} \\ &{}\times \biggl(\frac{1}{(2^{k} \cdot3l)^{n}} \int_{\widetilde {E}_{k}} \bigl\vert f(y) \bigr\vert ^{r'}\,dy \biggr)^{1/r'} \\ \lesssim{}& \sum_{ \vert \alpha \vert =m} \bigl\Vert D^{\alpha}b \bigr\Vert _{*} M_{r'}(f) (x)\lesssim \sum _{ \vert \alpha \vert =m} \bigl\Vert D^{\alpha}b \bigr\Vert _{*}M_{s}(f) (x_{0}) \end{aligned}$$

and

$$\begin{aligned} N_{142}\lesssim{}& \sum_{k=1}^{\infty}\bigl(2^{k} \cdot3l\bigr)^{n}\biggl(\frac {1}{(2^{k}\cdot3l)^{n}} \int_{\widetilde{E}_{k}} \bigl\vert K(x-y)-K(x_{1}-y) \bigr\vert ^{r}\,dy\biggr)^{1/r} \\ &{}\times\sum_{ \vert \alpha \vert =m}\biggl(\frac{1}{(2^{k}\cdot3l)^{n}} \int_{\widetilde {E}_{k}} \bigl\vert \bigl(D^{\alpha}b(y)- \bigl(D^{\alpha}b\bigr)_{\widetilde{F}_{k}}\bigr)f(y) \bigr\vert ^{r'}\,dy \biggr)^{1/r'} \\ \lesssim{}& \sum_{ \vert \alpha \vert =m} \bigl\Vert D^{\alpha}b \bigr\Vert _{*}M_{s}(f) (x_{0}). \end{aligned}$$

Finally, let us estimate $N_{2}$. Note that the integral

$$\begin{aligned} \int_{\mathbb{R}^{n}}\bigl(\chi_{\{\epsilon_{i+1}< \vert x-y \vert < \epsilon_{i}\} }(y)-\chi_{\{\epsilon_{i+1}< \vert x_{1}-y \vert < \epsilon_{i}\}}(y) \bigr) \frac{R_{m+1}({b};x_{1}-y)}{ \vert x_{1}-y \vert ^{m}}K(x_{1}-y)f_{2}(y)\,dy \end{aligned}$$

will only be non-zero if either $\chi_{\{\epsilon_{i+1}< \vert x-y \vert <\epsilon _{i}\}}(y)=1$ and $\chi_{\{\epsilon_{i+1}< \vert x_{1}-y \vert <\epsilon_{i}\}}(y)=0$ or vice versa. That means the integral will only be non-zero in the following cases:

(i) $\epsilon_{i+1}< \vert x-y \vert <\epsilon_{i}$ and $\vert x_{1}-y \vert \leq \epsilon_{i+1}$;
(ii) $\epsilon_{i+1}< \vert x-y \vert <\epsilon_{i}$ and $\vert x_{1}-y \vert \geq \epsilon_{i}$;
(iii) $\epsilon_{i+1}< \vert x_{1}-y \vert <\epsilon_{i}$ and $\vert x-y \vert \leq \epsilon_{i+1}$;
(iv) $\epsilon_{i+1}< \vert x_{1}-y \vert <\epsilon_{i}$ and $\vert x-y \vert \geq \epsilon_{i}$.

In case (i) we observe that $\epsilon_{i+1}< \vert x-y \vert \leq \vert x_{1}-x \vert + \vert x_{1}-y \vert <3l+\epsilon_{i+1}$ as $\vert x-x_{0} \vert < l$. Similarly, in case (iii) we have $\epsilon_{i+1}< \vert x_{1}-y \vert <3l+\epsilon _{i+1}$ as $\vert x-x_{0} \vert < l$. In case (ii) we have $\epsilon_{i}< \vert x_{1}-y \vert <3l+\epsilon_{i}$, and in case (iv) we have $\epsilon_{i}< \vert x-y \vert <3l+\epsilon_{i}$. By (1) we have

$$\begin{aligned} & \biggl\vert \int_{\mathbb{R}^{n}}\bigl(\chi_{\{\epsilon_{i+1}< \vert x-y \vert < \epsilon _{i}\}}(y)-\chi_{\{\epsilon_{i+1}< \vert x_{1}-y \vert < \epsilon_{i}\}}(y) \bigr) \frac{R_{m+1}({b};x_{1},y)}{ \vert x_{1}-y \vert ^{m}}K(x_{1}-y)f_{2}(y)\,dy \biggr\vert \\ &\quad\lesssim \int_{\mathbb{R}^{n}}\chi_{\{\epsilon_{i+1}< \vert x-y \vert < \epsilon_{i}\}}(y) \chi_{\{\epsilon_{i+1}< \vert x-y \vert < 3l+\epsilon_{i+1}\}}(y) \biggl\vert \frac{R_{m+1}({b};x_{1},y)}{ \vert x_{1}-y \vert ^{m}} \biggr\vert \frac { \vert f_{2}(y) \vert }{ \vert x_{1}-y \vert ^{n}}\,dy \\ &\qquad{}+ \int_{\mathbb{R}^{n}}\chi_{\{\epsilon_{i+1}< \vert x-y \vert < \epsilon_{i}\}}(y) \chi_{\{\epsilon_{i}< \vert x_{1}-y \vert < 3l+\epsilon_{i}\}}(y) \biggl\vert \frac{R_{m+1}({b};x_{1},y)}{ \vert x_{1}-y \vert ^{m}} \biggr\vert \frac { \vert f_{2}(y) \vert }{ \vert x_{1}-y \vert ^{n}}\,dy \\ &\qquad{}+ \int_{\mathbb{R}^{n}}\chi_{\{\epsilon_{i+1}< \vert x_{1}-y \vert < \epsilon_{i}\}}(y) \chi_{\{\epsilon_{i+1}< \vert x_{1}-y \vert < 3l+\epsilon_{i+1}\}}(y) \biggl\vert \frac{R_{m+1}({b};x_{1},y)}{ \vert x_{1}-y \vert ^{m}} \biggr\vert \frac { \vert f_{2}(y) \vert }{ \vert x_{1}-y \vert ^{n}}\,dy \\ &\qquad{}+ \int_{\mathbb{R}^{n}}\chi_{\{\epsilon_{i+1}< \vert x_{1}-y \vert < \epsilon_{i}\}}(y) \chi_{\{\epsilon_{i}< \vert x-y \vert < 3l+\epsilon_{i}\}}(y) \biggl\vert \frac{R_{m+1}({b};x_{1},y)}{ \vert x_{1}-y \vert ^{m}} \biggr\vert \frac { \vert f_{2}(y) \vert }{ \vert x_{1}-y \vert ^{n}}\,dy \\ &\quad= P_{1}+P_{2}+P_{3}+P_{4}. \end{aligned}$$

It is easy to check that $\vert x-y \vert \geq9l$, $\vert x_{1}-y \vert \geq8l$, and $\frac{8}{11} \vert x-y \vert \leq \vert x_{1}-y \vert \leq\frac{4}{3} \vert x-y \vert $ for $x\in B,y\in (10B)^{c}$; moreover, if $3l\geq\epsilon_{i+1},i\in\mathbb{N}$, we have

$$\begin{aligned} \bigl\{ y\in(10B)^{c}: \epsilon_{i+1}< \vert x-y \vert < 3l+\epsilon_{i+1}\bigr\} \subset\bigl\{ y\in (10B)^{c}: \vert x-y \vert < 6l\bigr\} =\phi \end{aligned}$$

and

$$\begin{aligned} \bigl\{ y\in(10B)^{c}: \epsilon_{i+1}< \vert x_{1}-y \vert < 3l+\epsilon_{i+1}\bigr\} \subset\bigl\{ y \in(10B)^{c}: \vert x-y \vert < 6l\bigr\} =\phi; \end{aligned}$$

this means $P_{1}=P_{3}=0$. Similarly, $P_{2}=P_{4}=0$ for $3l\geq\epsilon _{i}, i\in\mathbb{N}$. By Hölder’s inequality with *t* satisfying $1< t<\sqrt{\min(r',\rho)}$, we get

$$\begin{aligned} &P_{1}\lesssim\biggl( \int_{\mathbb{R}^{n}}\chi_{\{\epsilon _{i+1}< \vert x-y \vert < \epsilon_{i}\}}(y) \biggl\vert \frac{R_{m+1}({b};x_{1},y)}{ \vert x_{1}-y \vert ^{m}} \biggr\vert ^{t} \frac{ \vert f_{2}(y) \vert ^{t}}{ \vert x_{1}-y \vert ^{nt}}\,dy \biggr)^{1/t}\bigl[(3l+\epsilon _{i+1})^{n}-( \epsilon_{i+1})^{n}\bigr]^{1/t'}, \\ &P_{2}\lesssim\biggl( \int_{\mathbb{R}^{n}}\chi_{\{\epsilon _{i+1}< \vert x-y \vert < \epsilon_{i}\}}(y) \biggl\vert \frac{R_{m+1}({b};x_{1},y)}{ \vert x_{1}-y \vert ^{m}} \biggr\vert ^{t}\frac { \vert f_{2}(y) \vert ^{t}}{ \vert x_{1}-y \vert ^{nt}}\,dy \biggr)^{1/t}\bigl[(3l+\epsilon _{i})^{n}-( \epsilon_{i})^{n}\bigr]^{1/t'}, \\ &P_{3} \lesssim\biggl( \int_{\mathbb{R}^{n}}\chi_{\{\epsilon _{i+1}< \vert x_{1}-y \vert < \epsilon_{i}\}}(y) \biggl\vert \frac{R_{m+1}({b};x_{1},y)}{ \vert x_{1}-y \vert ^{m}} \biggr\vert ^{t}\frac { \vert f_{2}(y) \vert ^{t}}{ \vert x_{1}-y \vert ^{nt}}\,dy \biggr)^{1/t}\bigl[(3l+\epsilon _{i+1})^{n}-( \epsilon_{i+1})^{n}\bigr]^{1/t'} \end{aligned}$$

and

$$\begin{aligned} P_{4}\lesssim\biggl( \int_{\mathbb{R}^{n}}\chi_{\{\epsilon _{i+1}< \vert x_{1}-y \vert < \epsilon_{i}\}}(y) \biggl\vert \frac{R_{m+1}({b};x_{1},y)}{ \vert x_{1}-y \vert ^{m}} \biggr\vert ^{t}\frac { \vert f_{2}(y) \vert ^{t}}{ \vert x_{1}-y \vert ^{nt}}\,dy \biggr)^{1/t}\bigl[(3l+\epsilon _{i})^{n}-( \epsilon_{i})^{n}\bigr]^{1/t'}. \end{aligned}$$

Note that for $3l<\epsilon_{i+1}$, we have $(3l+\epsilon _{i+1})^{n}-(\epsilon_{i+1})^{n}\lesssim(\epsilon_{i+1})^{n-1}l$. Then

$$\begin{aligned} P_{1}\lesssim{}& \frac{(3l+\epsilon_{i+1})^{n}-(\epsilon_{i+1})^{n}}{(\epsilon _{i+1})^{(n-1)/t'}}\biggl( \int_{\mathbb{R}^{n}}\chi_{\{\epsilon _{i+1}< \vert x-y \vert < \epsilon_{i}\}}(y) \biggl\vert \frac{R_{m+1}({b};x_{1},y)}{ \vert x_{1}-y \vert ^{m}} \biggr\vert ^{t}\frac { \vert f_{2}(y) \vert ^{t}}{ \vert x_{1}-y \vert ^{nt}}\,dy \biggr)^{1/t} \\ \lesssim{}& l^{1/t'}\biggl( \int_{\mathbb{R}^{n}}\chi_{\{\epsilon _{i+1}< \vert x-y \vert < \epsilon_{i}\}}(y) \biggl\vert \frac{R_{m+1}({b};x_{1},y)}{ \vert x_{1}-y \vert ^{m}} \biggr\vert ^{t}\frac{ \vert f_{2}(y) \vert ^{t}}{ \vert x_{1}-y \vert ^{n+t-1}}\,dy \biggr)^{1/t}. \end{aligned}$$

Similarly, we have

$$\begin{aligned} &P_{2}\lesssim l^{1/t'}\biggl( \int_{\mathbb{R}^{n}}\chi_{\{\max\{\epsilon _{i+1},\frac{3}{4}\epsilon_{i}\}< \vert x-y \vert < \epsilon_{i}\}}(y) \biggl\vert \frac {R_{m+1}({b};x_{1},y)}{ \vert x_{1}-y \vert ^{m}} \biggr\vert ^{t}\frac{ \vert f_{2}(y) \vert ^{t}}{ \vert x_{1}-y \vert ^{n+t-1}}\,dy \biggr)^{1/t}, \\ &P_{3}\lesssim l^{1/t'}\biggl( \int_{\mathbb{R}^{n}}\chi_{\{\epsilon _{i+1}< \vert x_{1}-y \vert < \epsilon_{i}\}}(y) \biggl\vert \frac{R_{m+1}({b};x_{1},y)}{ \vert x_{1}-y \vert ^{m}} \biggr\vert ^{t}\frac{ \vert f_{2}(y) \vert ^{t}}{ \vert x_{1}-y \vert ^{n+t-1}}\,dy \biggr)^{1/t} \end{aligned}$$

and

$$\begin{aligned} P_{4}\lesssim l^{1/t'}\biggl( \int_{\mathbb{R}^{n}}\chi_{\{\max\{\epsilon _{i+1},\frac{8}{11}\epsilon_{i}\}< \vert x_{1}-y \vert < \epsilon_{i}\}}(y) \biggl\vert \frac {R_{m+1}({b};x_{1},y)}{ \vert x_{1}-y \vert ^{m}} \biggr\vert ^{t}\frac{ \vert f_{2}(y) \vert ^{t}}{ \vert x_{1}-y \vert ^{n+t-1}}\,dy \biggr)^{1/t}. \end{aligned}$$

Then

$$\begin{aligned} N_{2}= {}&\biggl\Vert \biggl\{ \int_{\mathbb{R}^{n}}\bigl(\chi_{\{\epsilon _{i+1}< \vert x-y \vert < \epsilon_{i}\}}(y)-\chi_{\{\epsilon_{i+1}< \vert x_{1}-y \vert < \epsilon _{i}\}}(y) \bigr) \\ &{} \times\frac {R_{m+1}({b};x_{1},y)}{ \vert x_{1}-y \vert ^{m}}K(x_{1}-y)f_{2}(y)\,dy \biggr\} _{\beta=\{\epsilon _{i}\}\in\Theta} \biggr\Vert _{F_{\rho}}\\ \lesssim {}& l^{1/t'} \biggl\Vert \biggl\{ \biggl( \int_{\mathbb{R}^{n}}\chi_{\{ \epsilon_{i+1}< \vert x-y \vert < \epsilon_{i}\}}(y) \biggl\vert \frac{R_{m+1}({b};x_{1},y)}{ \vert x_{1}-y \vert ^{m}} \biggr\vert ^{t}\frac{ \vert f_{2}(y) \vert ^{t}}{ \vert x_{1}-y \vert ^{n+t-1}}\,dy \biggr)^{1/t} \biggr\} _{\beta =\{\epsilon_{i}\}\in\Theta} \biggr\Vert _{F_{\rho}} \\ &{}+ l^{1/t'} \biggl\Vert \biggl\{ \biggl( \int_{\mathbb{R}^{n}}\chi_{\{\epsilon _{i+1}< \vert x_{1}-y \vert < \epsilon_{i}\}}(y) \biggl\vert \frac{R_{m+1}({b};x_{1},y)}{ \vert x_{1}-y \vert ^{m}} \biggr\vert ^{t}\frac{ \vert f_{2}(y) \vert ^{t}}{ \vert x_{1}-y \vert ^{n+t-1}}\,dy \biggr)^{1/t} \biggr\} _{\beta =\{\epsilon_{i}\}\in\Theta} \biggr\Vert _{F_{\rho}}\\ ={}& N_{21} +N_{22}. \end{aligned}$$

A straight computation deduces that

$$\begin{aligned} N_{21}\lesssim {}& l^{1/t'}\sup_{\epsilon_{i}\searrow0 }\biggl( \sum_{i} \biggl( \int_{\mathbb{R}^{n}}\chi_{\{\epsilon_{i+1}< \vert x-y \vert < \epsilon_{i}\}}(y) \biggl\vert \frac{R_{m+1}({b};x_{1},y)}{ \vert x_{1}-y \vert ^{m}} \biggr\vert ^{t}\frac{ \vert f_{2}(y) \vert ^{t}}{ \vert x_{1}-y \vert ^{n+t-1}}\,dy \biggr)^{\rho/t} \biggr)^{1/\rho}\\ \lesssim{}& l^{1/t'}\biggl( \int_{(10B)^{c}} \biggl\vert \frac {R_{m+1}({b};x_{1},y)}{ \vert x_{1}-y \vert ^{m}} \biggr\vert ^{t}\frac{ \vert f(y) \vert ^{t}}{ \vert x_{1}-y \vert ^{n+t-1}}\,dy\biggr)^{1/t} \\ \lesssim{}& l^{1/t'}\Biggl(\sum_{k=1}^{\infty}\int_{\widetilde{E}_{k}} \biggl\vert \frac{R_{m+1}(\widetilde{b}_{k};x_{1},y)}{ \vert x_{1}-y \vert ^{m}} \biggr\vert ^{t}\frac{ \vert f(y) \vert ^{t}}{ \vert x_{1}-y \vert ^{n+t-1}}\,dy\Biggr)^{1/t}. \end{aligned}$$

Similar to the estimates for $N_{13}$, for $x\in B, y\in\widetilde {E}_{k}$, we have

$$\begin{aligned} \bigl\vert R_{m+1}({\widetilde{b}}_{k};x_{1},y) \bigr\vert \lesssim \vert x_{1}-y \vert ^{m} \sum _{ \vert \alpha \vert =m}\bigl( \bigl\Vert D^{\alpha}b \bigr\Vert _{*}+ \bigl\vert D^{\alpha}b(y)-\bigl(D^{\alpha}b \bigr)_{\widetilde {F}_{k}} \bigr\vert \bigr). \end{aligned}$$

Then

$$\begin{aligned} N_{21}\lesssim{}& l^{1/t'}\sup_{\epsilon_{i}\searrow0 }\biggl( \sum_{i} \biggl( \int_{\mathbb{R}^{n}}\chi_{\{\epsilon_{i+1}< \vert x-y \vert < \epsilon_{i}\}}(y) \biggl\vert \frac{R_{m+1}({b};x_{1},y)}{ \vert x_{1}-y \vert ^{m}} \biggr\vert ^{t}\frac{ \vert f_{2}(y) \vert ^{t}}{ \vert x_{1}-y \vert ^{n+t-1}}\,dy \biggr)^{\rho/t} \biggr)^{1/\rho} \\ \lesssim{}& l^{1/t'}\biggl( \int_{(10B)^{c}} \biggl\vert \frac {R_{m+1}({b};x_{1},y)}{ \vert x_{1}-y \vert ^{m}} \biggr\vert ^{t}\frac{ \vert f(y) \vert ^{t}}{ \vert x_{1}-y \vert ^{n+t-1}}\,dy\biggr)^{1/t} \\ \lesssim{}& l^{1/t'}\Biggl(\sum_{k=1}^{\infty}\int_{\widetilde{E}_{k}}\sum_{ \vert \alpha \vert =m}\bigl( \bigl\Vert D^{\alpha}b \bigr\Vert ^{t}_{*}+ \bigl\vert D^{\alpha}b(y)-\bigl(D^{\alpha}b\bigr)_{\widetilde{F}_{k}} \bigr\vert ^{t}\bigr)\frac{ \vert f(y) \vert ^{t}}{ \vert x_{1}-y \vert ^{n+t-1}}\,dy \Biggr)^{1/t} \\ \lesssim{}& l^{1/t'}\sum_{ \vert \alpha \vert =m} \bigl\Vert D^{\alpha}b \bigr\Vert _{*}\sum_{k=1}^{\infty}\biggl( \int_{\widetilde{E}_{k}}\frac{ \vert f(y) \vert ^{t}}{ \vert x_{1}-y \vert ^{n+t-1}}\,dy \biggr)^{1/t} \\ &{} +l^{1/t'}\sum_{k=1}^{\infty}\biggl( \int_{\widetilde{E}_{k}}\sum_{ \vert \alpha \vert =m} \bigl\vert D^{\alpha}b(y)-\bigl(D^{\alpha}b\bigr)_{\widetilde{F}_{k}} \bigr\vert ^{t}\frac { \vert f(y) \vert ^{t}}{ \vert x_{1}-y \vert ^{n+t-1}}\,dy\biggr)^{1/t}. \end{aligned}$$

However,

$$\begin{aligned} &\sum_{k=1}^{\infty}\biggl( \int_{\widetilde{E}_{k}}\frac { \vert f(y) \vert ^{t}}{ \vert x_{1}-y \vert ^{n+t-1}}\,dy\biggr)^{1/t} \\ &\quad \lesssim \sum_{k=1}^{\infty}\frac{1}{(2^{k}\cdot3l)^{n/t+1/t'}}\biggl( \int _{ \vert x_{0}-y \vert < 2^{k+1}\cdot3l} \bigl\vert f(y) \bigr\vert ^{t}dt \biggr)^{1/t} \\ &\quad \lesssim M_{t}f(x_{0})\sum_{k=1}^{\infty}\frac{(2^{k}\cdot 3l)^{n/t}}{(2^{k}\cdot3l)^{n/t+1/t'}} \\ &\quad \lesssim l^{-1/t'}M_{r'}f(x_{0})\lesssim l^{-1/t'}M_{s}f(x_{0}) \end{aligned}$$

and

$$\begin{aligned} &\sum_{k=1}^{\infty}\biggl( \int_{\widetilde{E}_{k}}\sum_{ \vert \alpha \vert =m} \bigl\vert D^{\alpha}b(y)-\bigl(D^{\alpha}b\bigr)_{\widetilde{F}_{k}} \bigr\vert ^{t}\frac { \vert f(y) \vert ^{t}}{ \vert x_{1}-y \vert ^{n+t-1}}\,dy\biggr)^{1/t} \\ &\quad \lesssim\sum_{k=1}^{\infty}\frac{1}{(2^{k}\cdot3l)^{n/t+1/t'}}\biggl( \int _{\widetilde{E}_{k}}\sum_{ \vert \alpha \vert =m} \bigl\vert D^{\alpha}b(y)-\bigl(D^{\alpha}b\bigr)_{\widetilde{F}_{k}} \bigr\vert ^{t} \bigl\vert f(y) \bigr\vert ^{t} \,dy \biggr)^{1/t} \\ &\quad\lesssim \sum_{k=1}^{\infty}\frac{1}{(2^{k}\cdot3l)^{n/t+1/t'}}\biggl( \int _{\widetilde{F}_{k}} \bigl\vert f(y) \bigr\vert ^{t^{2}} \,dy \biggr)^{1/t^{2}} \biggl(\sum_{ \vert \alpha \vert =m} \int_{\widetilde{F}_{k}} \bigl\vert D^{\alpha}b(y)- \bigl(D^{\alpha}b\bigr)_{\widetilde{F}_{k}} \bigr\vert ^{t't} \,dy \biggr)^{1/(tt')} \\ &\quad \lesssim \sum_{ \vert \alpha \vert =m} \bigl\Vert D^{\alpha}b \bigr\Vert _{*} M_{r'}f(x_{0}) \sum _{k=1}^{\infty}\frac{(2^{k}\cdot3l)^{n/t^{2}+1/(tt')}}{(2^{k}\cdot 3l)^{n/t+1/t'}} \\ &\quad = \sum_{ \vert \alpha \vert =m} \bigl\Vert D^{\alpha}b \bigr\Vert _{*} M_{r'}f(x_{0}) \sum_{k=1}^{\infty}\frac {(2^{k}\cdot3l)^{n/t}}{(2^{k}\cdot3l)^{n/t+1/t'}} \\ &\quad \lesssim l^{-1/t'}\sum_{ \vert \alpha \vert =m} \bigl\Vert D^{\alpha}b \bigr\Vert _{*} M_{s}f(x_{0}). \end{aligned}$$

Then

$$\begin{aligned} N_{21}\lesssim\sum_{ \vert \alpha \vert =m} \bigl\Vert D^{\alpha}b \bigr\Vert _{*} M_{s}f(x_{0}). \end{aligned}$$

Similarly,

$$\begin{aligned} N_{22}\lesssim\sum_{ \vert \alpha \vert =m} \bigl\Vert D^{\alpha}b \bigr\Vert _{*} M_{s}f(x_{0}). \end{aligned}$$

This completes the proof of Theorem 1.

## Conclusion

In this paper, we have established the weighted $L^{p}$-boundedness of variation and oscillation operators for a family of multilinear singular integrals with kernels satisfying certain Hörmander type conditions. These results extend the corresponding work in [9] and [15].

## References

[1] Bougain J. (1989). Pointwise ergodic theorem for arithmetic sets. Publ. Math. Inst. Hautes Études Sci..

[2] Campbell J.T., Jones R.L., Reinhold K., Wierdl M. (2000). Oscillation and variation for the Hilbert transform. Duke Math. J..

[3] Campbell J.T., Jones R.L., Reinhold K., Wierdl M. (2003). Oscillation and variation for singular integrals in higher dimension. Trans. Am. Math. Soc..

[4] Gillespie T.A., Torrea J.L. (2004). Dimension free estimates for the oscillation of Riesz transforms. Isr. J. Math..

[5] Crescimbeni R., Macías R.A., Menárguez T., Torrea J.L., Viviani B. (2009). The *ρ*-variation as an operator between maximal operators and singular integrals. J. Evol. Equ..

[6] Ma T., Torrea J.L., Xu Q. (2015). Weighted variation inequalities for differential operators and singular integrals. J. Funct. Anal..

[7] Ma T., Torrea J.L., Xu Q. (2017). Weighted variation inequalities for differential operators and singular integrals in higher dimensions. Sci. China Math..

[8] Lorente M., Riveros M.S., de la Torre A. (2005). Weighted estimates for singular integral operators satisfying Hörmander’s conditions of Young type. J. Fourier Anal. Appl..

[9] Zhang J., Wu H.X. (2017). Weighted oscillation and variation inequalities for singular integrals and commutators satisfying Hörmander type condition. Acta Math. Sin. Engl. Ser..

[10] Cohen J. (1981). A sharp estimate for a multilinear singular integral on $\mathbb{R}^{n}$. Indiana Univ. Math. J..

[11] Cohen J., Gosselin J. (1986). A *BMO* estimate for multilinear singular integral operators. Ill. J. Math..

[12] Ding Y., Lu S.Z. (2001). Weighted boundedness for a class rough multilinear operators. Acta Math. Sin..

[13] Lu S.Z., Wu H.X., Zhang P. (2003). Multilinear singular integral with rough kernel. Acta Math. Sin..

[14] Hu G.E., Yang D.C. (2000). A variant sharp estimate for multilinear singular integral operators. Stud. Math..

[15] Hu Y., Wang Y.S. (2017). Oscillation and variation inequalities for the multilinear singular integrals related to Lipschitz functions. J. Inequal. Appl..

[16] John J., Nirenberg L. (1961). On functions of bounded mean oscillation. Commun. Pure Appl. Math..

[17] Muckenhoupt B. (1972). Weighted norm inequalities for the Hardy maximal function. Trans. Am. Math. Soc..

